# Comparative discourse analysis of logical memory recall in normal elderly from rural and urban areas

**DOI:** 10.1590/1980-5764-DN-2024-0164

**Published:** 2025-02-03

**Authors:** Lúcia Inês de Araújo, Sérgio Leme da Silva, Leonardo Caixeta

**Affiliations:** 1 Universidade Federal de Goiás, Hospital das Clínicas, Serviço de Fonoaudiologia, Goiânia GO, Brazil.; 2 Universidade Federal de São Carlos, Laboratório de Neurociências do Bem-Estar, Departamento de Psicologia, São Carlos SP, Brazil.; 3 Universidade Federal de Goiás, Faculdade de Medicina, Hospital das Clínicas, Departamento de Neurologia, Serviço de Neurologia Cognitiva e Neuropsiquiatria (CERNE), Goiânia GO, Brazil.

**Keywords:** Memory, Short-Term, Wechsler Memory Scale, Rural Areas, Urban Area, Memória de Curto Prazo, Escala de Memória de Wechsler, Zona Rural, Área Urbana

## Abstract

This study examined the nature of errors in prose recall made in normal aging from different cultural backgrounds: rural and urban areas.

**Objective:**

The aim of this study was to examine the nature of errors in prose recall made in normal aging from different cultural backgrounds.

**Methods:**

We evaluated neurologically normal elderly divided into two groups: 12 subjects resident in a rural area in Central Brazil (group 1, called “Interior”) and 10 subjects from a metropolitan area in a big city in Central Brazil (group 2, called “Metropolis”). Responses by 22 nondemented older adults to the Logical Memory subtest of the Wechsler Memory Scale were examined in a propositional analysis.

**Results:**

Healthy older adults from rural areas (interior group) showed good immediate recalls, but had deficits in retention over a delay. Subjects from urban areas made errors of omission, not commission, at immediate recall.

**Conclusion:**

These errors probably reflect difficulty with attentional control rather than memory per se. There are differences in the performance of the elderly when considering different levels of education for neuropsychological tests associated with immediate recalls (but not with delayed recall), suggesting that working memory systems may be more impacted by years of schooling than the coding memory system.

## RESUMO

Este estudo examinou a natureza dos erros na recordação de texto no envelhecimento normal para diferentes origens culturais: áreas rurais e urbanas.

**Objetivo:**

Examinar a natureza de erros na recordação de textos em idosos normais de diferentes culturas.

**Métodos:**

Foram avaliados idosos neurologicamente normais divididos em dois grupos: 12 sujeitos residentes em área rural do Brasil Central (grupo 1, denominado “Interior”), e dez sujeitos provenientes de região metropolitana de uma grande cidade do Brasil Central (grupo 2, denominado “Metrópole”). As respostas apresentadas por 22 idosos não demenciados para o subteste Memória Lógica da Escala de Memória de Wechsler foram corrigidos em uma análise proposicional.

**Resultados:**

Idosos saudáveis moradores de área rural (grupo interior) tiveram boa recordação imediata, mas apresentaram déficits na retenção para recordação tardia. Sujeitos moradores de área urbana cometeram erros de omissão na recordação imediata.

**Conclusão:**

Esses erros provavelmente refletem dificuldades com o controle da atenção, em vez da memória em si. Existem diferenças no desempenho dos idosos ao se considerarem diferentes níveis de ensino para testes neuropsicológicos associados à memória imediata (mas não com a recordação tardia), o que sugere que o sistema de memória de trabalho pode ser mais afetado por anos de escolaridade do que o sistema de memória de codificação.

## INTRODUCTION

The assessment of memory can take many forms, and there are several tests to assess memory in its different segments. One of the most used is the Logical Memory of Wechsler Memory Scale (WMS)^1^. The logical memory subtest is a good tool to differentiate healthy older adults from those who supposedly can be in the early stages of dementia, with a view that recent memory difficulties are common at this stage^2^. Notwithstanding its translation and adaptation of extreme care, several researchers noted that the criteria for original correction of this scale are not very specific when the person sends a partially correct answer to the correction unit, giving rise to subjectivity in judging the correct answers^3,4^. Due to these results, some authors suggested the use of another way to evaluate the results of this test to eliminate ambiguities in the criteria of correctness, called propositional analysis. This analysis consists of decomposing the text into small units of language, called “propositions,” that hold meaning in context. Propositions contain interconnections, which give meaning to the text material. Thus, the elements of propositions are concepts of the text, rather than specific words of the same. Proposition is therefore understood as being the smallest unit of speech that still has meaning; thus, the exact contents of a single proposition can vary, depending on its use, but the lexicon and the semantic relationships among its elements do not change^1^.

Cross-cultural neuropsychology is concerned with the investigation of the influence of culture and its mechanisms on cognitive functions, as well as the modeling that cultural variables have on neuropsychological symptoms of disease. It also highlights the need to customize the measurement instruments — often imported — used in the neuropsychological assessment in ethnically and culturally distinct populations^5^.

Within-group variation is an important yet understudied component of cross-cultural neuropsychology. Luria conducted a series of field studies on the neuropsychological performance of individuals living in villages and very isolated and undeveloped rural areas of Uzbekistan and Kyrgyzstan (Central Asia)^6^. In this same work, Luria described that illiterate rural workers, living in these areas, had performed in a less elaborate way than the subjects with higher education and other types of occupancy (less menial and more intellectual tasks). Those in the first group did not use the same logic for deductions when compared to the second group, nor established relationships between the objects equally, and they used less abstraction. To prepare their responses often evoked their everyday experiences, these responses referring more to what they knew in fact (concrete reasoning) than to abstractions, inferences, and hypotheses about the phenomena (deductive reasoning)^6^. In 1984, Gilbert performed a replication of Luria’s Uzbek study in rural Kwa Zulu, a province of South Africa, with near-identical results^7^.

In this paper, we analyze the cognitive performance of neurologically healthy elderly from different cultural and educational backgrounds in the logical memory subtest to describe potential cultural and educational factors that can modulate neuropsychological answers in different scenarios.

The aim of this study was to examine the nature of errors in prose recall made in normal aging from different cultural backgrounds.

## METHODS

We evaluated neurologically normal elderly of both genders, more than 60-years-old, divided into two groups of seniors: 12 subjects resident in a rural area in Central Brazil — countryside of Teresina de Goiás, in Goiás State (group 1, called “Interior”) and 10 subjects from a metropolitan area in a city in Central Brazil — Goiânia, capital of Goiás (group 2, called “Metropolis”).

Subjects without autonomy, dependent on caregivers, or who complained of decreased hearing and visual acuity were discarded because of the linguistic and visual nature of the tests. The experimental procedures were approved by the Ethics Committee of the University of Brasília (086/2008). All participants signed a written informed consent agreement prior to participation in the study.

To be considered “normal” subjects, one must score according to the mini mental state examination (MMSE) and Katz^8^ functional questionnaire.

Inclusion criteria: For the metropolitan group, individuals must be over 60 years of age, have autonomy identified in the anamnesis and Katz index, have lived in a metropolitan city environment for more than 30 consecutive years, and have a level of education and occupation compatible with the inner city group (low education, no intellectual occupation, or manual labor). In the interior group, subjects must be over the age of 60 years, with autonomy as shown by anamnesis and Katz index, living in the surroundings of Teresina de Goiás since birth.

### Experimental procedures

All sessions were carried out in a predetermined place, in a controlled setting, in which there were no external stimuli to distract the attention of the subjects taking part in the study. Before the sessions, all participants took a strict anamnesis/clinical interview, a detailed questionnaire answered by the subject and/or accompanying person, in which the minimal conditions to take part were investigated; correction follows Spreen and Strauss^9^.

### Neuropsychological tests

The MMSE developed by Folstein et al.^10^ verifies, through tasks, the temporal–spatial orientation, immediate memory, attention, and calculus, recall memory, and language. It involves issues regarding naming the days of the week, objects, and mathematical operations, translated and adapted by Bertolucci^11^.

WMS subtest II — short-term and long-term memory assessment; the subtests are: information (important for observing remote and immediate recall as well as for the content assessment of the sense of space and time, the date, month, and year in which the subject thinks he/she is at the moment of test); mental control (which involves simple logical reasoning), attention, and free recall (immediate memory). Logical memory I and II — involves immediate verbal recall and late recall (30 minutes late), respectively. It ensures that the subject can memorize a verbal context of both short-term and long-term stories^12^. We also make use of contextualized stories for the metropolis group and the elderly living in a rural area, with the same number of passages from WMS Subtest II. This test is recommended by the Brazilian Academy of Neurology^13^.

During data collection, subjects were told three stories, two (A and B) of which belonged to WMS, subtests I and II, and another one was made up out of the individual’s reality.

Then, the subjects were asked to immediately recall each story they had been told. After 30 min, they were required to try to recall as much as they could about each story. Importantly, subjects did not show awareness of late recall.

The assessment of the outcomes consisted of decomposing the text into small units of language, termed utterances, which retain meaning within the context. Propositions contain interconnections, which provide meaning to the material of the text. In this manner, the elements of propositions are textual concepts, rather than specific text words. It is worth noting that, what is understood as a proposition is the smallest unit of the discourse that conserves meaning; thus, the exact content of a unique proposition may vary, depending on its use, yet lexicon and semantic relations among their elements remain unchanged^1^.

### Statistical analyses

Statistics were assessed by the SPSS 12.0 software, having begun with the test of differences of average and variance (ANOVA) to verify whether there were any significant differences among groups, as well as if there was significance among original stories narrated from WMS and the story adapted to the reality of the subject. The procedure used for comparing both Interior and Metropolis groups was the Student’s *t*-test.

### Procedures

During data collection, three stories were read to the subject: two, A and B, belonging to WMS, subtest II, call: logical memory I literal correction (LM1LC), logical memory I propositional correction (LM1PC), logical memory II literal correction (LM2LC), and logical memory II correction propositional (LML2PC), both in immediate and late recall, as in the latter we added T, for instance, logical memory I late correction late recall literal correction (LM1TLC). The third story created from the individual’s reality — literal correction — is called contextualized logical memory literal correction (LMConTLC), contextualized logical memory as well, adding T to late recall, for example, contextualized late logical recall (LMContT).

It was then prompted for the individuals to instantly hear every story. After 30 min, they were asked again to try to remember as many of the stories as they could. Subjects were unaware of the task of delayed recall. Student’s t-test, commonly used to test equality between two means, was applied to compare the Interior group with the Metropolis group.

## RESULTS

The p<0.5 as shown in Table 1 and indicated by (*) shows significant differences between both groups; the respective t-value (absolute value) found was higher than the standard t-value under the level of 5% of significance; thus, we refuse the hypothesis that the compared groups have equal performance, i.e., there are differences concerning performance in the elderly group when the following is applied.

**Table 1:** Performance of the elderly group in the Wechsler Memory Scale Subtest II neuropsychological tests.

Wechsler Memory Scale Subtest I and II	Groups		Student’s *t*-test	p-value
	Interior (n=12)	Metropolis (n=10)	calc.	
LM1LC	1.75	7.70	4.82	0.00*
SD	2.56	3.23		
LM1PC	3.25	10.90	4.00	0.00*
SD	4.56	4.36		
LM1TLC	0.50	1.10	0.82	0.41
SD	1.73	1.66		
LM1TPC	1.08	1.90	0.64	0.52
SD	3.45	2.23		
LM2LC	1.17	7.20	3.70	0.00*
SD	1.85	4.87		
LM2PC	2.25	9.40	3.77	0.00*
SD	3.22	5.56		
LM2TLC	0.25	1.50	1.50	0.16
SD	0.87	2.50		
LM2TPC	0.25	2.60	1.77	0.10
SD	0.87	4.11		
LMConTLC	3.25	7.90	3.42	0.00*
SD	3.22	3.11		
LMContPC	5.50	10.40	2.57	0.01*
SD	5.02	3.66		
LMContTLC	2.00	2.50	0.39	0.70
SD	3.41	2.42		
LMContTPC	4.08	3.90	0.09	0.93
SD	5.75	3.81		

Abbreviations: SD, standard deviation; LM1LC, Logical Memory I Literal Correction; LM1PC, Logical Memory I Propositional Correction; LM1TLC, Logical Memory I Late Recall Literal Correction; LM1TPC, Logical Memory I Late Recall Propositional Correction; LM2LC, Logical Memory II Literal Correction; LM2PC, Logical Memory II Correction Propositional; LM2TLC, Logical Memory II Late Recall Literal Correction; LM2TPC, Logical Memory II Late Recall Correction Propositional; LMConTLC, Logical Memory Contextualized Literal Correction; LMContPC, Logical Memory Contextualized Correction Propositional; LMConTLC, Logical Memory Contextualized Late Recall Literal Correction; LMContTPC, Logical Memory Contextualized Late Recall Correction Propositional.

Note: Student’s *t-*test=2.09 for the 5% level of statistical significance.

*shows significant differences between both groups.

The procedure used to compare groups concerning the educational levels in Table 2 was the analysis of variance. The p<0.05 presented in Table 2 and indicated by (*) shows that there are significant differences between the groups considered and means followed by the same letters do not statistically differ among themselves at the level of 5% of significance. Thus, we reject the hypothesis that different levels of education do not produce different neuropsychological performances, that is, there are differences in the performance of groups of elderly people when considering different levels of education for the following neuropsychological tests: LM1Lit, LM1Prop, and LM2Prop.

**Table 2:** Performance of elderly in neuropsychological tests divided into groups of educational levels.

Wechsler Memory Scale Subtest I and II:	Years of schooling			F-test	p-value
	[0–3]	[4 ou +]	[8 ou +]		
LM1LC	2.18^a^	6.88^b^	6.33^ab^	4.43	0.02*
SD	3.46	3.64	4.04		
LM1PC	3.09^a^	9.88^b^	11.67^b^	6.73	0.00*
SD	4.37	5.08	4.93		
LM1TLC	0.36^a^	1.63^a^	0.00a	1.78	0.19
SD	0.81	2.50	0.00		
LM1TPC	0.55^a^	3.13^a^	0.33^a^	2.32	0.12
SD	1.21	4.29	0.58		
LM2LC	1.82^a^	5.63^a^	7.00a	2.73	0.09
SD	2.60	5.60	5.57		
LM2PC	2.64^a^	7.88^ab^	9.67b	3.68	0.04*
SD	3.64	6.40	5.69		
LM2TLC	0.09^a^	1.50^a^	1.67a	1.80	0.19
SD	0.30	2.50	2.89		
LM2TPC	0.36^a^	2.13^a^	2.67^a^	1.16	0.33
SD	0.81	4.16	4.62		
LMContLC	3.82^a^	7.13^a^	6.33a	1.93	0.17
SD	3.57	4.39	1.15		
LMContPC	6.09^a^	9.38^a^	9.33a	1.19	0.32
SD	5.01	5.55	1.15		
LMContTLC	1.45^a^	2.75^a^	3.67a	0.85	0.44
SD	2.38	3.49	3.51		
LMContTPC	2.73^a^	5.25^a^	5.33a	0.74	0.49
SD	3.74	5.92	6.11		

Abbreviations: SD, standard deviation; LM1LC, Logical Memory I Literal Correction; LM1PC, Logical Memory I Propositional Correction; LM1TLC, Logical Memory I Late Recall Literal Correction; LM1TPC, Logical Memory I Late Recall Propositional Correction; LM2LC, Logical Memory II Literal Correction; LM2PC, Logical Memory II Correction Propositional; LM2TLC, Logical Memory II Late Recall Literal Correction; LM2TPC, Logical Memory II Late Recall Correction Propositional; LMConTLC, Logical Memory Contextualized Literal Correction; LMContPC, Logical Memory Contextualized Correction Propositional; LMConTLC, Logical Memory Contextualized Late Recall Literal Correction; LMContTPC, Logical Memory Contextualized Late Recall Correction Propositional.

Note: Standard F-test=3.52 to the 5% level of statistical significance.

*shows that there are significant differences between the groups considered.

## DISCUSSION

The current study explored the potential source of variation in a neurologically healthy Brazilian elderly sample by comparing the neuropsychological test performance of nondemented groups of urban and rural elders who live in Central-West Brazil. Age, education, and gender are the most common covariates used to define normative standards against which neuropsychological performance (NP) is interpreted, but influences of other demographic factors have begun to be appreciated. In developing nations, urban versus rural residence may differentially affect numerous factors that could influence cognitive test performances, including the quality of both formal and informal educational experiences and employment opportunities. Such disparities may necessitate corrections for urban/rural (U/R) status in NP norms^14^. The results of our study reveal a significant difference between groups regarding Logical Memory subtests I and II in Literal and Propositional correction and Logical Memory subtest with the contextualized story also in the two types of correction.

The results of our study show similar performances between the groups with regard to the Grammatical Support Index in both boards 01 and 02, as well as in the Delayed Logical Memory (in literal and propositional corrections). About the stories in context, the scores show the importance of contextualization of neuropsychological tests, since this procedure has approximated group performances.

In this study, it was possible to find which tests were not influenced by educational level: Logical Memory Late Recall (Propositional and Literal Correction) and Contextualized Logical Memory Late Recall (Literal and Propositional Correction). These tests seem to be suitable and therefore eligible for use in neuropsychological studies with low education samples. Concerning the contextualized stories, the scores reveal the importance of contextualization of neuropsychological assessment materials, since the interior group approached the Metropolis. This study highlights the need for more research that can build protocols for analyzing mnemonic cognition in the elderly when originating from samples that are very different from those that originated from conventional test regulations and aligned with the pharmaceutical industry. The data presented here align with several authors^6,15-18^ and research focusing on ecological approaches to understanding the functional conditions appropriate to rural realities. This perspective emphasizes the importance of utilizing contextual stimuli and alternative corrections to address the cultural backgrounds and educational deficits of these individuals. Additionally, it raises questions about the functional difficulties faced by this population in real-world situations and their reasoning abilities^15^, that is, distinct cutoff standards for practical and spatial cognition and conceptual reasoning cognition. Discoveries about these demands can assist in a more efficient clinical pharmacological prescription and the possibility of creating a more differential protocol for memory disorders for diagnosing dementia with higher levels of sensitivity and specificity than the cognitive screening regulations used today when using the MMSE, highly criticized when it comes to uneducated, rural, and culturally specific elderly people.

When we take into account the qualitative analysis of WMS subtest II, it is evidenced that the elderly individuals in the metropolis group reached the cutoff point in both literal and propositional correction, according to Hodges^16^, for immediate free memory recovery, i.e., having 25% of items recalled. At the same time, the elderly of the interior group almost reached this percentage, but only under the condition of contextualized stories and propositional correction in logical memory. Regarding the qualitative analysis for logical memory (late recovery), the two groups reached the cutoff point of normality, by following the aforementioned author. We found that the contextualized logical memory test is eligible for use in the evaluation of illiterate subjects having different cultural background, since it enables the real identification of memory deficits. The analysis of the late recall logical memory test showed that propositional correction is more efficient since it is not influenced by educational status.

The analysis of cultural differences can help not only to discern the influence of cultural background on neuropsychological test performance but may also contribute to a better understanding of the cerebral organization of cognitive activity^17^. It may be the case in our sample that processes of modernization changed traditional ways of thinking, i.e., people from urban areas are more exposed to selective cognitive pressure with greater demands on immediate memory processes than people from rural areas, and thus they have developed to a greater degree abilities that deal with working memory issues. It seems that subjects from poor rural areas and illiterate constantly use more practical intelligence and spatial than conceptual reasoning; it seems they act and build, rather than stand in the cognitive–analytic world of ideas.

Initial correlational research suggested that tests evaluating memory for word lists versus stories were essentially interchangeable^18^. Both require rapid processing of a constant stream of information that must be maintained in working memory to facilitate encoding and consolidation via hippocampally based mechanisms. Patients with focal frontal lobe lesions^19^ and executive dysfunction^20^ exhibit worse memory for unrelated words than semantically related information. Conversely, memory for semantically related information, including stories, may be more directly related to temporal lobe integrity^21,22^.

The main limitation of this study is due to the small number of participants. The data was obtained from a larger ongoing study, which main focus is on the neuropsychological aspects of individuals’ residents in isolated communities. The difficult access to these communities is partially responsible for this small number.
